# Spatio-Temporal Super-Resolution Reconstruction of Remote-Sensing Images Based on Adaptive Multi-Scale Detail Enhancement

**DOI:** 10.3390/s18020498

**Published:** 2018-02-07

**Authors:** Hong Zhu, Xinming Tang, Junfeng Xie, Weidong Song, Fan Mo, Xiaoming Gao

**Affiliations:** 1 Satellite Surveying and Mapping Application Center, NASG, Beijing 100048, China; zhuhong@sasmac.cn (H.Z.); txm@sasmac.cn (X.T.); mof@sasmac.cn (F.M.); gaoxm@sasmac.cn (X.G.); 2 College of Resource Environment and Tourism, Capital Normal University, Beijing 100048, China; 3 Key Laboratory of Satellite Surveying and Mapping Technology and Application, NASG, Beijing 10048, China; 4 School of Earth Science and Engineering, Hohai University, Nanjing 211100, China; 5 School of Surveying and Geographical Science, Liaoning Technical University, Fuxin 123000, China; lntu_swd@163.com

**Keywords:** remote-sensing image, super-resolution reconstruction, multi-scale deposed, adaptive detail enhancement

## Abstract

There are many problems in existing reconstruction-based super-resolution algorithms, such as the lack of texture-feature representation and of high-frequency details. Multi-scale detail enhancement can produce more texture information and high-frequency information. Therefore, super-resolution reconstruction of remote-sensing images based on adaptive multi-scale detail enhancement (AMDE-SR) is proposed in this paper. First, the information entropy of each remote-sensing image is calculated, and the image with the maximum entropy value is regarded as the reference image. Subsequently, spatio-temporal remote-sensing images are processed using phase normalization, which is to reduce the time phase difference of image data and enhance the complementarity of information. The multi-scale image information is then decomposed using the *L*_0_ gradient minimization model, and the non-redundant information is processed by difference calculation and expanding non-redundant layers and the redundant layer by the iterative back-projection (IBP) technique. The different-scale non-redundant information is adaptive-weighted and fused using cross-entropy. Finally, a nonlinear texture-detail-enhancement function is built to improve the scope of small details, and the peak signal-to-noise ratio (PSNR) is used as an iterative constraint. Ultimately, high-resolution remote-sensing images with abundant texture information are obtained by iterative optimization. Real results show an average gain in entropy of up to 0.42 dB for an up-scaling of 2 and a significant promotion gain in enhancement measure evaluation for an up-scaling of 2. The experimental results show that the performance of the AMED-SR method is better than existing super-resolution reconstruction methods in terms of visual and accuracy improvements.

## 1. Introduction

We address the problem of generating a high-resolution (HR) image given multiple low-resolution (LR) images [1,2]. At present, this is a research hotspot in the remote-sensing image processing field. In the super-resolution reconstruction technology applied in optical satellite remote-sensing image processing, homologous or heterogeneous sequences of remote-sensing images with the same area are used for super-resolution (SR) reconstruction to improve image spatial resolution and image quality [3,4,5]. SR reconstruction technology can enhance the spatial resolution of satellite imagery at a lower economic cost by making full use of satellite remote-sensing image data without increasing hardware investment. Broadly speaking, the core idea of super-resolution reconstruction is to increase the spatial resolution by using the time bandwidth (acquiring the multi temporal image sequence of the same scene), which is to achieve the transformation from temporal resolution to spatial resolution. Remote-sensing image data of this paper were acquired from multi-platforms, multi-temporal, multi-viewpoints, which can be considered as a kind of spatio-temporal remote-sensing image. Spatio-temporal remote-sensing image can provide non-redundant information, enhance complementary information in spatial domain, and improve texture-feature representation. Consequently, it is an effective method for the super-resolution reconstruction to take advantage of the important information provided by the spatio-temporal remote-sensing image. Thus, the utilization efficiency of remote-sensing image data can be realistically improved. So, the research results have important theoretical significance and practical value [6]. Currently, SR reconstruction methods can be generally divided into four categories [7]:

(1) *Image interpolation* [8,9]: This field has been extensively studied, and the studies show that image interpolation is not only flexible but also calculationally fast. However, image interpolation is inherently limited since it is based on local grayscale values of low-resolution images to estimate grayscale information of unknown pixels. Therefore, the lost or degraded high-frequency information cannot be recovered during the image interpolation process. Which is perhaps caused by image edge diffusion to different degrees or due to the phenomenon of high-frequency information blurring found in reconstructed images.
(2) *Reconstruction-based techniques* [10,11,12,13]: These approaches require accurate prior knowledge for SR reconstruction, but prior knowledge of remote-sensing images with complex topography is still difficult to acquire [14].
(3) *Learning-based techniques* [15,16]: Learning-based techniques estimate high-frequency details from a large training set of HR images that encode the relationship between HR and LR images. These techniques required a large training set. The missing high-frequency detail information of the reconstruction image is supplemented based on similarities between the LR image and the HR image in the training set. Recently, there have been the state-of-the-art SR method to be put forward. Dong et al. [17] introduced image super-resolution using deep convolutional networks (SRCNN). Kim et al. [18] proposed accurate image super-resolution using very deep convolutional networks (VDSR). These approaches have shown great promise. Owing to the fact that the texture of a remote-sensing image is complex, the training process is time-consuming, and it is challenging task to achieve real-time processing in practical engineering.
(4) *Enhancement-based techniques* [19,20]: These approaches estimate an SR image using image enhancement on the up-sampled image and require an image enhancement method technique that increases the loss of high-frequency information and improves the effect of image reconstruction. The cited studies focus on how to increase the detail information as well as on how representation schemes can be conducted in such spaces. In the pioneering work of Vishnukumar et al. [21], a single-image SR technique for remote-sensing images using content-adaptive, detail-enhanced self-examples was proposed. Sun et al. [22] introduced a gradient profile prior to the reconstruction image when performing single-image SR and sharpness enhancement. Yu et al. [23] put forward an image SR approach based on gradient enhancement. Local constraints are established to achieve an enhanced gradient map, while the global sparsity constraints are imposed on the gradient field to reduce noise effects in SR results.

To summarize, SR reconstructions using these methods have resulted in a certain degree of progress and breakthroughs. They solve the problem of the complexity of the SR reconstruction model, but their drawback is that the edge of the reconstructed image is possibly over-sharpened due to the insufficiency of the image-enhancement method. They also ignore the difference information between the series of images. Meanwhile, these methods are not adaptive for different kinds of image contents. Being quite different from those previous methods, in this paper we investigate the SR problem from an information enhancement viewpoint and propose a joint SR method based on adaptive multi-scale detail enhancement.

## 2. Adaptive Multi-Scale Detail-Enhancement Image SR

**Figure 1:**
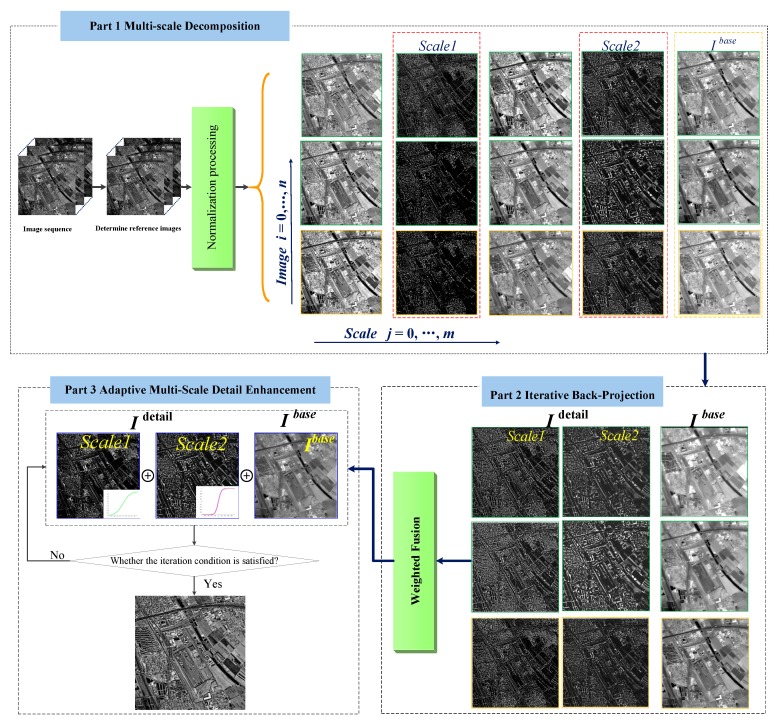
The framework of AMDE-SR.

In view of existing reconstruction-based SR algorithms, existing texture representation is not sufficient and high-frequency details are lacking. This paper proposes a novel SR method based on adaptive multi-scale detail enhancement based on spatio-temporal remote-sensing images with complementary information. An adaptive detail-enhancement method is applied to SR reconstruction to improve the reconstruction image quality, and highlight the detail features of the HR image. In addition, we extend our work to make the reconstructed image clearer, the edge structure more obvious, and the high-frequency information more abundant. Hence, our method is relatively accurate and fast compared to state-of-the-art methods. Figure 1 shows the entire AMDE-SR process flow. Experimental results on a variety of remote-sensing image sequences show that the proposed method can largely improve the quality of SR results, and increase the high-frequency information of HR images.

### 2.1. Spatio-Temporal Remote-Sensing Data Preprocessing

To make full use of complementary information between the spatio-temporal remote-sensing images, and make the SR reconstruction images contain much more texture-detail information, we process the registered spatio-temporal remote-sensing images. The well-performing image registration method reported in [24] is used in this paper. On the basis of images registered, we determine a reference image among spatio-temporal remote-sensing images through information entropy. Spatio-temporal remote-sensing images are represented as *I*_1_, …, *I_t_*. The entropy of each remote-sensing image is computed used the entropy function. *P*(*x_i_*) is the probability of appearing *x_i_* (*i* = 1, 2,…, *n*), where *x_i_* represents the gray level of pixels, and the entropy function is described as Equation (1). Then the information entropy of each spatio-temporal remote-sensing image is compared, and the maximum entropy is selected as the reference image:

(1) $$Q(x)\equiv \sum_{i=1}^{n}P(x_{i})\log_{2}\left(\frac{1}{P(x_{i})}\right)=-\sum_{i=0}^{n}P(x_{i})\log_{2}P(x_{i})$$

Next, we must normalize the spatio-temporal remote-sensing data. This process can fully leverage the advantages of spatio-temporal remote-sensing image data for high temporal resolution, reduce the differences between spatio-temporal remote-sensing data, and enhance the complementarity of spatio-temporal remote-sensing data. This step is completed by the least-squares method. Supposing that *I*_1_, *I*_2_, …, *I_t_* satisfies the function relation *f*, in order to allow spatio-temporal remote-sensing image data to be closer to the reference image *I_ref_*, phase normalization is done by the adjustment method, and the mathematical description is as shown by:

(2) $$I_{ref}=f_{i}(I_{i})+\varepsilon_{i},i\in [1,t]$$

where ε represents the residuals between spatio-temporal image data, and *f_i_* represents an affine function, such as *I_ref_ = a_i_I_i_ + b_i_*, where *a_i_* and *b_i_* are transformation parameters corresponding to spatio-temporal remote-sensing data, and the least square is used to solve the transformation parameters. When the residual error is a minimum, the normalization of the spatio-temporal remote-sensing data is complete.

### 2.2. L*_0_* Gradient Minimization Model

In this paper, the multi-scale decomposition of normalized images is performed using the *L*_0_ gradient minimization model. Xu et al. [25] introduced a robust method with *L*_0_ gradient minimization, which can achieve a global optimum by processing the whole image. The number of non-zero gradients is confined in the method of *L*_0_ gradient minimization in order to enhance the contrast the utmost. *L*_0_ gradient minimization is based on two constraints: one is that a smoothed image must be very close to the original input image, and the other is that the image must be flat after image smoothing. These two constraints are used to perform the modeling, for which the formula is:

(3) $$\underset{S}{\min}\{\sum_{p}{\left(S_{p}-I_{p}\right)}^{2}+\lambda \cdot C(S)\}$$

where *S_p_* is the output image after smoothing, *I_p_* is the input image, and *p* is a pixel index here. The function *C* is a sparse gradient counting tool for image smoothing, which is defined as follows:

(4) $$C(S)=\#\{p|\left|\partial_{h}S_{p}\right|+\left|\partial_{v}S_{p}\right|\neq 0\}$$

In practice, the function *C*(*S*) is used to calculate the sum of the pixels in the vertical and horizontal method of smoothing images. *λ* is a variable which controls the cumulative relationship between the former formula and the *C*(*S*). *S* in Equation (4) is the desired image, $\left|\partial_{h}S_{p}\right|$ and $\left|\partial_{v}S_{p}\right|$ are gradients in the horizontal and vertical directions of image *S*, and #{} is a counter, which is used to output the number pixels of $\left|\partial_{h}S_{p}\right|+\left|\partial_{v}S_{p}\right|\neq 0$. Because the function *C*(*S*) has no convex optimization problem, the formula for the function deformation is:

(5) $$\underset{S,h,v}{\min}\{\sum_{p}{\left(S_{p}-I_{p}\right)}^{2}+\lambda C(h,v)+\beta ({\left(\partial_{x}S_{p}-h_{p}\right)}^{2}+{\left(\partial_{v}S_{p}-v_{p}\right)}^{2})\}$$

where $C(h,v)=\#\left\{p|\left|h_{p}\right|+\left|v_{p}\right|\neq 0\right\}$, variables *h* and *v* indicate the vertical and horizontal gradients, respectively, and these two gradients are an approximation of the *S* gradient in Equation (5). *β* represents the adapted parameter, which controls the similarity between the corresponding gradients and the (*h*, *v*). The function deformation equation is divided into two parts. The first is *S*, using gradient descent to obtain results, while the second is the process of solving a smooth progression of *h* and *v*, which needs more discussion. Discussion of two kinds of situations has minimum value in calculating the value of *h* and *v*.

In the first step: computing *S*. The solution of *S* can be converted to the following terms. Xu et al. proposed using Fast Fourier Transform (FFT) to accelerate, written as:

(6) $$S=F^{-1}\left(\frac{F(I)+\beta (F{\left(\partial_{x}\right)}^{*}F(h)+F{\left(\partial_{y}\right)}^{*}F(v))}{F(1)+\beta (F{\left(\partial_{x}\right)}^{*}F(\partial_{x})+F{\left(\partial_{y}\right)}^{*}F(\partial_{y}))}\right)$$

where *F* is the Fast Fourier Transform operator, *F*()* is the complex conjugate. *F*(1) denotes the function of the Fourier Transform.

In the second step: computing *h* and *v*. The objective function of the step is described as Equation (7):

(7) $$\underset{h,v}{\min}\left\{\sum_{p}({\left(\partial_{x}S_{p}-h_{p}\right)}^{2}+{\left(\partial_{y}S_{p}-v_{p}\right)}^{2})+\frac{\lambda }{\beta }C(h,v)\right\}$$

In Equation (7), *C*(*h,v*) can be spatially decomposed to *h_p_* and *v_p_* and estimated individually. Therefore, Equation (7) can be converted to the following formula:

(8) $$\sum_{p}\underset{h_{p},v_{p}}{\min}\left\{{\left(h_{p}-\partial_{x}S_{p}\right)}^{2}+{\left(v_{p}-\partial_{y}S_{p}\right)}^{2}+\frac{\lambda }{\beta }H\left(\left|h_{p}\right|+\left|v_{p}\right|\right)\right\}$$

where *H* is a binary function, if $\left|h_{p}\right|+\left|v_{p}\right|\neq 0$, *H* returns 1, otherwise, *H* returns 0. In order to reaches the minimum value, the solution need to be discussed under the condition:

(9) $$(h_{p},v_{p})=\{\begin{array}{cc} \left(0,0\right) & (\partial_{x}S_{p}{)}^{2}+{\left(\partial_{y}S_{p}\right)}^{2}\leq \lambda /\beta \\ \left(\partial_{x}S_{p},\partial_{y}S_{p}\right) & \mathrm{otherwise} \end{array}$$

In this paper, the *L*_0_ gradient minimization model was used to perform multi-scale decomposition, for the aim of obtaining more detail information.

### 2.3. Multi-Scale Decomposition and Non-Redundant Spatial Information Extraction

The initial high-resolution image is reconstructed by the IBP method. The traditional IBP method presented in [26] is used in the AMED-SR method. In this paper, the initial reconstructed HR image is decomposed into multiple scales based on the *L*_0_ gradient minimization model. Setting different filtering parameters, the image is decomposed into a series of smooth layers with different scales. Here, we illustrate the process with an example, specifically a reference image. For the reference image, the target of the multi-scale decomposition is to acquire a set of smoothed images $s^{j}$ (scale *j* = 0, 1, …, *m*). Suppose that the reference image is decomposed into an *m*-layer smooth image. The image then must be decomposed at *j* + 1 levels (scale *j* = 0, 1, …, *m*). When the smoothed scale *j* = 0, we set *s*^0^ = *I^ref^*. Then, the *L*_0_ gradient minimization model was iteratively applied to the input image and a series of differently scaled smooth layers $s^{1},\cdots s^{j}$ are computed. The progressive smoothed by the different parameter *λ* increases the spatial smoothing at each level *j*. we set the smoothed scale parameter *λ_s=_*_1_ = 1 × 10^−3^, then set $\lambda_{s=j}=2^{j-1}\lambda_{s=1}$ for all *j* > 1. The other input image can be generated in the same manner. *s*^(*i,j*)^ denotes the largest-scale smooth layer of spatio-temporal remote-sensing image. Differential processing is carried out on the smooth layer of the adjacent two scales, and then differently scaled detail layers are obtained, the specific formula is:

(10) $$d^{\left(i,j\right)}=s^{\left(i,j-1\right)}-s^{\left(i,j\right)}$$

where:

(11) j=0,1,⋯,m; i=0,1,⋯,t

### 2.4. Non-Redundant Information Weighted Fusion

In this paper, the *L*_0_ gradient minimization model is utilized to perform multi-scale decomposition of remote-sensing image sequences. The ultimate goal of this process is to provide non-redundant information. On the basis of non-redundant spatial information extraction, different scales of non-redundant information should be weighted fused. To take advantage of the non-redundant information, cross-entropy is carried out to realize weighted fusion, rather than simply average fusion. Cross-entropy can measure the difference between the reference image and the input images’ information, so the cross-entropy is regarded as the weight value with which to calculate the difference information in the non-redundant spatial information. $d_{ref}=\left\{d_{ref}^{0},d_{ref}^{1},\cdots ,d_{ref}^{n}\right\}$ and $d_{img}=\left\{d_{img}^{0},d_{img}^{1},\cdots ,d_{img}^{n}\right\}$ represent grayscale probability distribution of the reference image and of the input remote-sensing images, respectively. The mathematical description of cross entropy is:

(12) $$\omega^{i}=\sum_{i=0}^{n}d_{ref}^{i}\log_{2}\frac{d_{ref}^{i}}{d_{img}^{i}}$$

If the cross-entropy is small, it means the differences between the reference image and input images are small; that is, the non-redundant information is less available in the process of reconstruction, the proportions of the weight of the fusion will be smaller, and vice versa. According to the correlation between the reference image and input images, the fusion weight value is determined by cross-entropy. The weight parameter *w* is introduced to realize the weighted fusion. For clarity, the same-scale information of the reference image and input images is calculated based on the weighted value *w^i^* across the cross-entropy. The multi-scale detail layer of different spatio-temporal remote-sensing image *d*^(*i,j*)^ is weighted fused as *d^j^*, which is the spatial frequency and contain the texture information in different scales. The non-redundant information weighted fusion can be written as:

(13) $$d^{j}=\frac{\sum_{i=1}^{n}\omega^{i}d^{\left(i,j\right)}}{\sum_{i=1}^{n}\omega^{i}}$$

### 2.5. Nonlinear Detail-Enhancement Function

After different scale information undergoes weighted fusion, we focus on the problem of high-frequency detail-information promotion. Here, the motivation of defining a nonlinear detail-enhancement function is that the cognition of the human visual system is a process extending from coarse to fine. First, the saliency characteristics of images are observed, such as color, brightness, contrast, etc. Second, the image texture structure, edge features, and other important detail information will be observed. From a biological neuroscience point of view, if we convert an image into a signal, the middle part of the image signal resembles the excited state of the neuron, and the edge region resembles the inhibitory state of neurons. Therefore, normalized image information, simulating the neuron processing of signals, defines the nonlinear detail-enhancement function, as expressed in Equation (14) below. This function enhances the small- and medium-scale detail information in the multi-scale decomposition process, and increases the high-frequency information in the reconstructed image:

(14) $$f(\beta ,d^{j})=(2/(1+\exp(-\beta *d^{j})))-1$$

**Figure 2:**
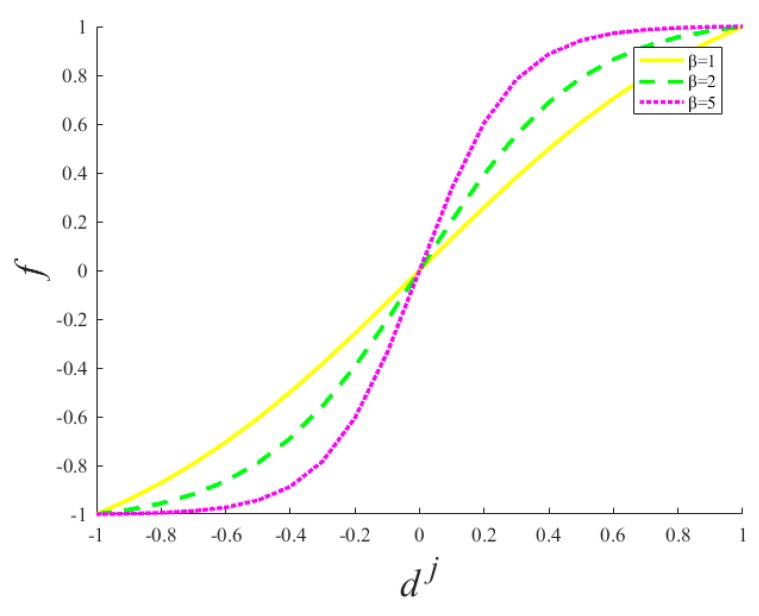
Detail-enhancement function of different parameters.

$f(\beta ,d^{j})$ in Equation (14) is the nonlinear detail-enhancement function, $\beta *d^{j}$ a simple scalar multiplication, parameter *β* a positive number in the detail-enhancement function, and *d^j^* the high-resolution differently scaled detail information. Figure 2 shows the detail-enhancement function of different parameters. From the Figure 2, we can see clearly that the high-frequency information provided by the function *f* is significantly increasing, with the increase of parameter *β*.

Details of the nonlinear detail-enhancement function are discussed next. The partial derivative of the nonlinear detail-enhancement function can be derived as:

(15) $$\frac{\partial }{\partial d^{j}}f(\beta ,d_{}^{j})=\frac{1}{2}\beta (1-f_{}^{2}(\beta ,d^{j}))$$

Thus, when *β* > 0, it can be proved that $f(\beta ,d^{j})$ is the increasing function of *d^j^*. In other words, the larger the parameter *β*, the more the detail information of the image will increase. In the SR reconstruction process, the quality index of the peak signal-to-noise–ratio is used as an iterative constraint condition to control the value of the parameter *β*. To achieve better reliability in reconstructing the SR image, local iterative optimization is carried out to realize adaptive multi-scale detail enhancement, and the iterative formula can be expressed as:

(16) $$\Delta (I_{HR},I_{ref})=\frac{1}{MN}{\left\|sP(\sum_{j}^{m}d^{j}+s^{j})-P(I_{ref})\right\|}_{2}^{2}$$

In Equation (16), *M*, *N* are the local window sizes, *s* the down-sampling factor, *I_HR_* the reconstructed image, *I_ref_* the reference image, and *P* the function of the PSNR. The primary purpose of iterative optimization is to acquire the high-resolution image with rich high-frequency information.

## 3. Experimental Results and Discussion

In this section, a variety of areas with different topographies are used in super resolution reconstruction experiments, in order to test the reliability and effectiveness of the proposed AMED-SR method. The experimental data come from different temporal and different sensors. The areas of remote-sensing images cover mountainous area, road, plain area, and city building area. In simulation experiment, input LR images are simulated by down sample by convoluting the real HR images. In real experiment, input LR images are the original satellite remote-sensing image.

### 3.1. Quantitative Evaluation Factors

For the sake of evaluating the quality of the super-resolution reconstruction results in our experimental, the following four classical quantitative evaluation factors are chosen in super resolution reconstruction field. In the simulation SR experiments, the full reference evaluation factors are selected, such as peak signal-to-noise ratio (PSNR) [27] and structural similarity index (SSIM) [28]. These reference quality evaluations require the original HR as reference image. In real experiments, we use the no reference image evaluation factors: entropy [29] and enhancement measure evaluation (EME) [30], by reason of the real HR image does not exist.

**Peak Signal-to-Noise Ratio (PSNR)**. In the field of super-resolution reconstruction, PSNR is one of the commonly quantitative evaluation method and mainly used to evaluate the degree of image distortion. In the quality evaluation of the super-resolution reconstruction results, the mean square error between the real HR image and the reconstructed HR image is computed. The higher PSNR value is, the better reconstruction image will be. The description of this index can be expressed as follows:

(17) $$PNSR=10\mathrm{lg}\frac{L^{2}mn}{\sum_{i=1}^{m}\sum_{j=1}^{n}{\left[I_{HR}(i,j)-I_{SR}(i,j)\right]}^{2}}$$

where $I_{HR}(i,j)$ is the real HR image and $I_{SR}(i,j)$ is the SR reconstruction image, *m* and *n* represent the line number and column number of the image, respectively, and *L* generally represents the gray distribution range of image. 

#### 3.1.1. Structural Similarity index (SSIM)

It is widely used in the super-resolution reconstruction quality evaluation. Wang et al. [28] introduced the structural similarity index, and the mathematical description of the SSIM index is defined as:

(18) $$SSIM=\frac{\left(2\mu_{x}\mu_{y}+C_{1})(2\delta_{xy}+C_{2}\right)}{\left(\mu_{x}^{2}+\mu_{y}^{2}+C_{1})(\delta_{x}^{2}+\delta_{y}^{2}+C_{2}\right)}$$

where *μ_x_*, *μ_y_* is the average of *x* and *y* respectively, $\delta_{x}^{2}$, $\delta_{y}^{2}$ are the variance of *x* and *y* respectively, $\delta_{xy}$ is the covariance of *x* and *y*, *C*_1_, *C*_2_ are the constants.

#### 3.1.2. Entropy

Entropy is used to represent the degree of uniform distribution of any energy in space. If the energy distribution is uniform better, the entropy value will be larger. The entropy of image information can be generally expressed as in Equation (1). The larger of the entropy value is, the more information of the image will be contained.

#### 3.1.3 Enhancement Measure Evaluation (EME)

The principle of enhancement measure evaluation is to calculate the maximum and minimum ratio of the gray level in the sub region, which is obtained by dividing the evaluated image into *k*_1_ × *k*_2_ sub regions. The logarithm of ratio is the evaluation result of the image details. This evaluation index represents the degree of gray change of the image local. The larger the EME value is, the richer the detail information of the image will have. Its mathematical expression is shown in Equation (19):

(19) $$EME_{k_{1},k_{2}}=\frac{1}{k_{1},k_{2}}\sum_{i=1}^{k_{2}}\sum_{k=2}^{k_{1}}20\log\frac{I_{\max;k,j}^{w}}{I_{\min;k,j}^{w}}$$

where $I_{\max;k,j}^{w}$, $I_{\min;k,j}^{w}$ denotes the maximum and minimum values of the local image blocks *w_k_*,*_l_*, respectively.

### 3.2. Simulation Image Experiments

**Figure 3:**
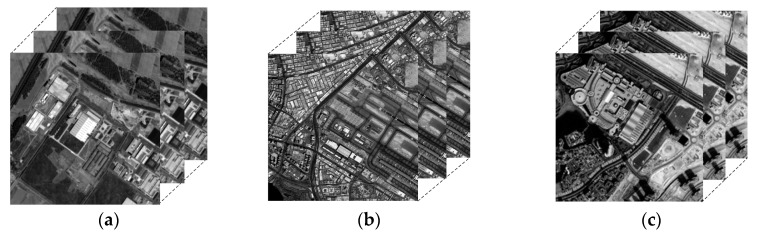
Simulated remote-sensing image data.

**Figure 4:**
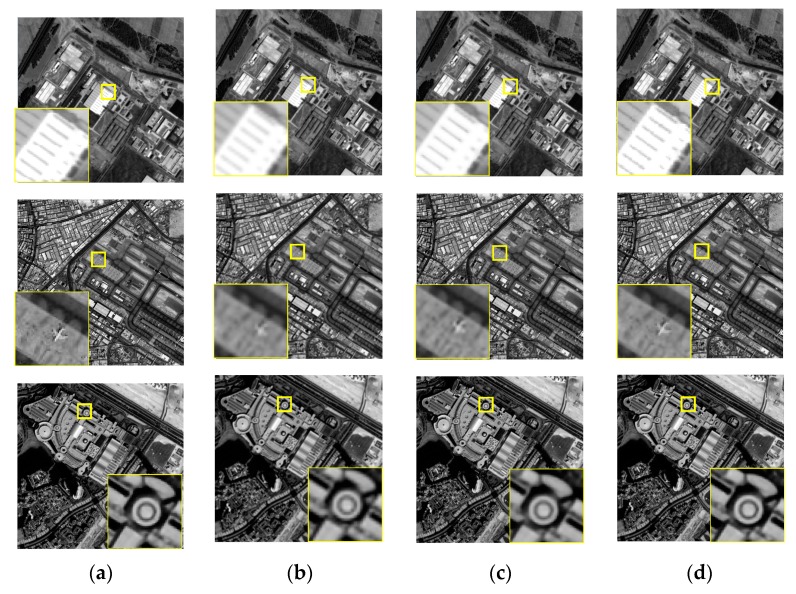
Simulation results of ZY-3 satellite image (**a**) Original remote-sensing image; (**b**) Bicubic; (**c**) SRCNN; (**d**) AMDE-SR.

It is difficult to obtain a real HR remote sensing image from the same sensor. Therefore, the effectiveness of the proposed super-resolution reconstruction method is verified by simulation experiments, and the original HR remote sensing images were obtained from ZY-3. Consideration length of thesis, we just select three simulated experiments to illustrate. The LR sequence image data with sub-pixel displacement relation is calculated through the simulation model *g_m_* = *K* * *f_m_*, where *f_m_* is the original high-resolution image by similarity transformation simulation operation, K the fuzzy convolution kernel, * representative convolution operation, *g_m_* the down-sampled image sequences. We compare the proposed method with other typical and state-of-the-art super resolution reconstruction methods. SR experiment with the scaling factor *s* = 2, Figure 3 and Figure 4 give the simulated remote-sensing image, and super resolution reconstruction results of different methods, respectively. In Figure 3, remote-sensing image comes from the resources satellite three (ZY-3), which is a series of surveying and mapping remote-sensing satellites. At present, ZY3-01 and ZY3-02 are in-orbit operation. Now, the network of ZY3-01 and ZY3-02 is operated to ensure the long-term stable acquisition of high-resolution remote-sensing data. In our SR experimental, the resolution of the ZY3-01 panchromatic nadir image is 2.1 m. The imagery in Figure 3 was taken on 6 June 2016, 21 September 2015 and 9 April 2013, respectively.

It can be seen from the experimental results, the edge structure of the interpolation SR result using simulated ZY-3 satellite imagery is blurry. Because the high-frequency information is lost in the SR reconstruction process and the difference between the edge structure and the smooth information is ignored. The SRCNN is one of the state-of-the-art SR methods, and the SR result is got through deep learning network structure. The edge structure is better than the bicubic method. The quality of the reconstructed image has also been improved significantly. The deficiency in the SRCNN method is that the texture information is still not enough. In comparison with experimental results through different SR methods, the MADE-SR method can retain a better edge structure, and the texture information is increased by the nonlinear detail enhancement function. In Figure 4, the edge of the house is clearly visible in the first experiment, the outline of a plane is more obvious in the second experiment and the edge structure of the building is clearer in the third experiment. That is, the edge structure is clearer and texture detail is supplemented in the proposed SR method of this paper.

**Figure 5:**
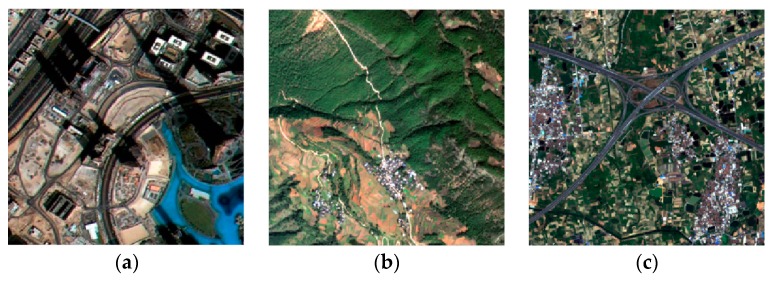
Simulation multi-temporal image (**a**) ZY-3 multi-temporal image; (**b**) GF-2 multi-temporal image; (**c**) WorldView-2 multi-temporal image.

**Figure 6:**
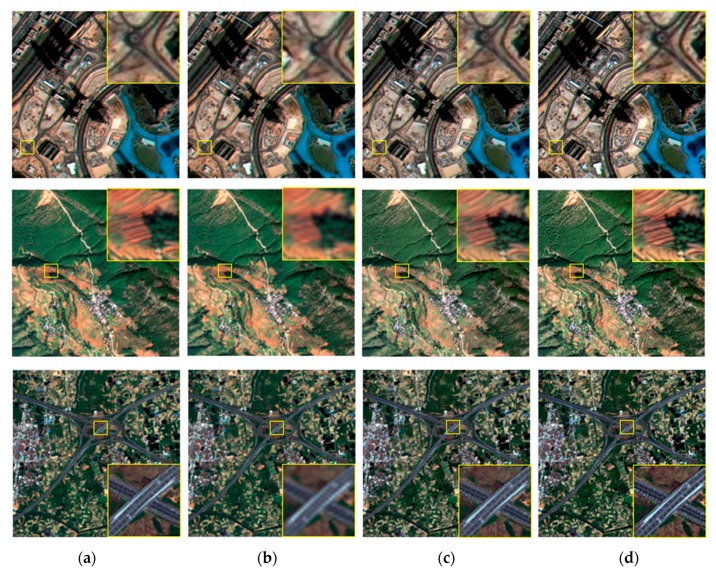
Simulation results of multi-spectral image (**a**) Original remote-sensing image; (**b**) Bicubic; (**c**) SRCNN; (**d**) AMDE-SR.

Remote-sensing satellites can obtain single band panchromatic images and multiband multi-spectral images at the same time. Thus, multi-spectral image is also one of the representative remote-sensing images. In the simulation experiment, the multi-spectral image is used to verify the effectiveness of the MADE-SR method. We choose the multi-spectral image from different sensors, such as ZY3-01, Gaofen-2 satellite (GF-2) and worldview-2. The resolution of the ZY3-01 multi-spectral image is 2.1 m. The imagery in Figure 5a was taken on 10 January 2017. The resolution of the GF-2 multi-spectral image is 3.2 m. The imagery in Figure 5b was taken on 11 November 2017. The resolution of the WorldVeiw-2 multi-spectral image is also 1.8 m. The imagery in Figure 5c was taken on 16 October 2017. In the multi-spectral image SR experimental, the red, green and blue band was selected. The three bands of multi-spectral image in the experiment are considered as the image with shorter interval which is taken from different CCD cameras. Then using the complementary information between bands to realize super-resolution reconstruction. We determine a reference band among multi-spectral image through entropy. The next process of the super-resolution reconstruction is the same as the panchromatic image. The simulated multi-spectral image is shown in Figure 5, and the SR result is shown in Figure 6.

The simulated multi-spectral image is calculated through the simulation model *g_m_* = *K* * *f_m_*, using the complementary information of multispectral spectral segments to fulfil the SR experiment. Figure 6 clearly shows that the MADE-SR method can protect the edge structure and include rich texture detail information.

In the simulated experiment, for the sake of evaluating the reconstruction results more objectively, the objective evaluation index of the PSNR and the SSIM are chosen to evaluate the SR images. The reconstructed images of the simulated experiments are presented in Figure 4 and Figure 6. We can see that the whole image blur-based bicubic, that is, the SR image based on interpolation method cannot increase the high-frequency information. Also, in Figure 6, we can see the SRCNN method applied to remote-sensing images, the edge of the SR images structures tends to a little blur, which is not effective in preserving the large-scale edges of remote-sensing image. In contrast, the SR images of the proposed method have better texture performance. The results of the reference quality assessment reveal that the SR images of the proposed method has better perform with respect to the image objective evaluation factors. The objective evaluation results of the different SR algorithms are listed in Table 1.

### 3.3. Real Remote-Sensing Image Experiments

**Figure 7:**
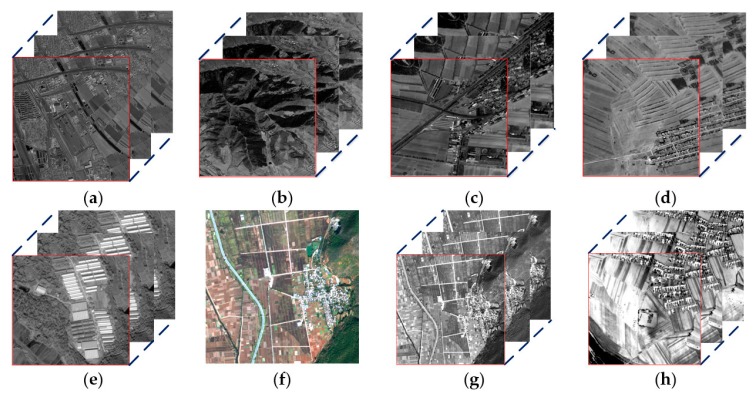
Experimental images. (**a**) city building area; (**b**) mountain area; (**c**) road area; (**d**) plain area; (**e**) plant area; (**f**) farmland area; (**g**) different band of (**f**); (**h**) village area.

The image data in real remote-sensing image experiments comes from a remote-sensing satellite. For real remote-sensing image, both the panchromatic image with different dates and the multi-spectral image with different bands belong to spatio-temporal remote-sensing image. Both of these images can provide complementary information in the process of super-resolution reconstruction. But the difference is that the time interval of the panchromatic image is relatively long while the imaging time of the multi-spectral images in different bands is relatively short. In real remote-sensing image experiments, the date of the multi-temporal panchromatic image is selected from similar dates. The reason is that the continuous covered panchromatic image from similar dates can provide complementary information without significant changes in topography. The reconstructed results of these real data sets are shown in Figure 7. The specific parameters of these imagery are listed in Table 2.

In Table 2, the imagery in Figure 7a was taken on the same day, but the time taken by the three CCD camera was actually different. The panchromatic imagery only has one band. The red, green and blue band of the multi spectral imagery is selected in the real remote-sensing SR experiment, it is shown in Figure 7g.

**Figure 8:**
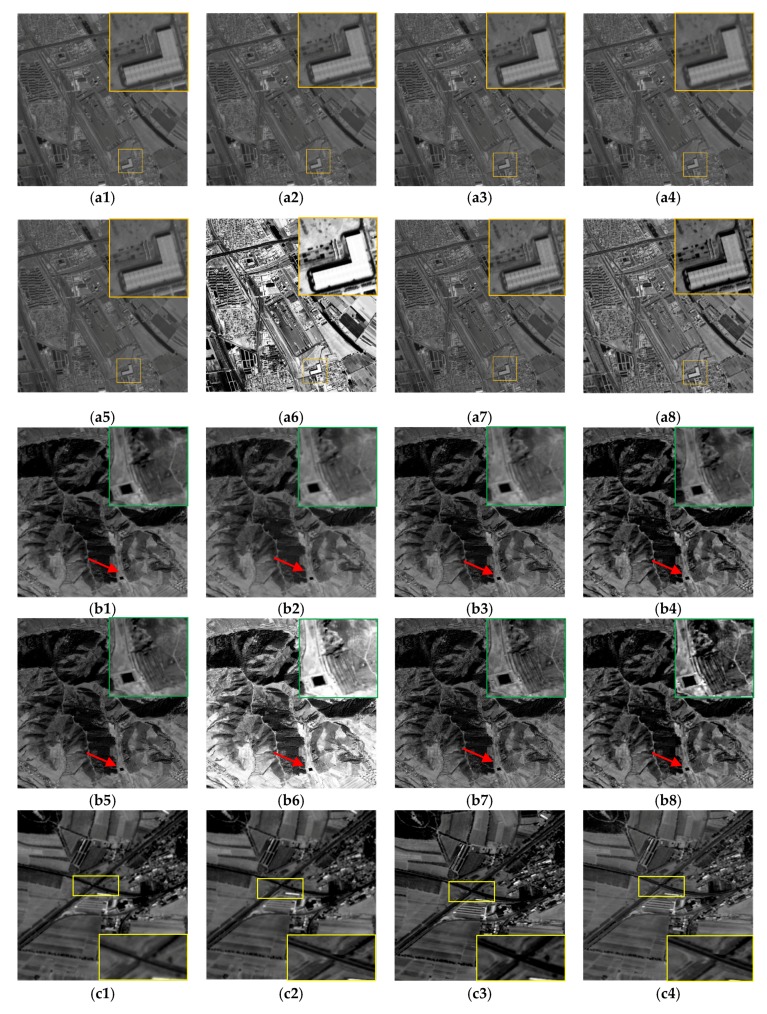

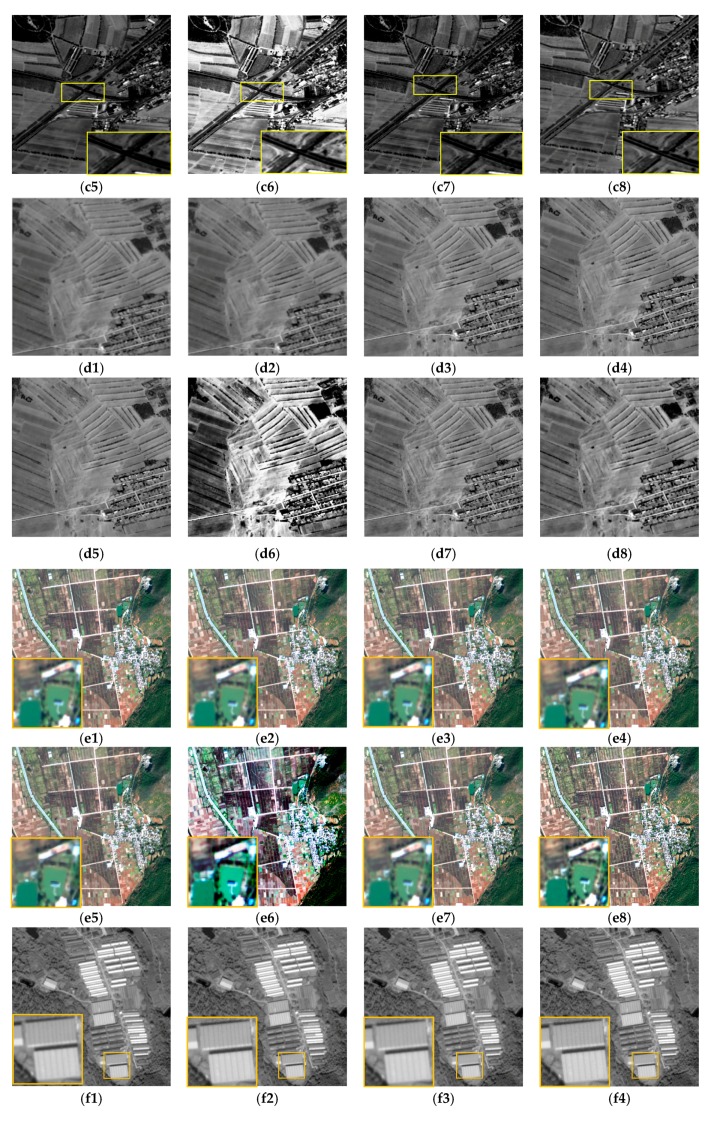

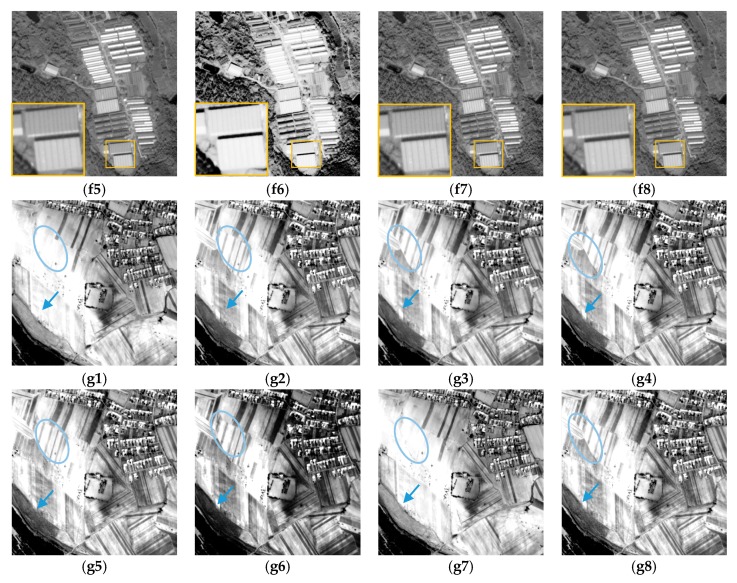
Reconstructed HR images of different areas by different super resolution methods. (**a1**,**b1**,**c1**,**d1**,**e1**,**f1**,**g1**) Bicubic; (**a2**,**b2**,**c2**,**d2**,**e2**,**f2**,**g2**) IBP; (**a3**,**b3**,**c3**,**d3**,**e3**,**f3**,**g3**) MAP; (**a4**,**b4**,**c4**,**d4**,**e4**,**f4**,**g4**) SRCNN; (**a5**,**b5**,**c5**,**d5**,**e5**,**f5**,**g5**) VDSR; (**a6**,**b6**,**c6**,**d6**,**e6**,**f6**,**g6**) HE; (**a7**,**b7**,**c7**,**d7**,**e7**,**f7**,**g7**) average fusion; (**a8**,**b8**,**c8**,**d8**,**e8**,**f8**,**g8**) MADE-SR.

In the real remote-sensing image experiment, experimental images of the first four groups come from the same sensor, and the acquisition time of the image was different. We compare the AMED-SR method with the traditional and the state-of-the-art SR methods, including bicubic method, IBP method [29], MAP method [14], SRCNN method [17] and VDSR method [18]. The results show that the definition of the reconstructed image based on interpolation method is not good. In Figure 8(a1,b1,c1,d1,e1,f1,g1), those reconstructed edge structures are also blurry. The IBP method and MAP method are in the range of the reconstructed SR reconstruction approach. The experimental results are shown in Figure 8(a2,b2,c2,d2,e2,f2,g2,a3,b3,c3,d3,e3,f3,g3).

The edge structure of reconstructed image is better than interpolation approach. However, the detail information is not prominent enough on the basis of IBP and MAP method. In addition, SRCNN and VDSR method are regarded as subordinate to the state-of-the-art SR method. A large number of experimental samples need to be trained in these deep learning SR methods, and the quality of the reconstructed image has been significantly improved. Nevertheless, the state-of-the-art reconstructed results often need to sacrifice a great deal of time. Furthermore, the promotion of the texture information is still limited. Moreover, to test independently contributions, we also compare the proposed method with SR result based on histogram equalization (HE) and SR result with the information average fusion. Compared to the reconstructed image in Figure 8(g7), the global of the SR image seems to miss the inherent information. Histogram equalization does not improve the local details of the reconstructed image. Meanwhile, the edge structure has not been well preserved. From here it can seen that the proposed MADE-SR method is not a simple contrast enhancement method. In the proposed method of this paper, the detail information of different scales can be improved by the nonlinear detail enhancement function. Contrast to the nonlinear detail enhancement function, there is no special change in visual of the reconstructed image through non-redundant information average fusion. In the proposed method of this paper, the contribution of the nonlinear detail enhancement is larger than the non-redundant information weighted fusion. But the objective of the weighted fusion is to make full use of the non-redundant information in spatio-temporal image. Because each image itself has a different amount of information as well as the contribution of each image to the reconstructed result is different (Table 3). To verify that the proposed method is applicable to different sensors, the remote-sensing images from different sensors are used in the experiment. The experimental results are shown in the next three groups in Figure 8 which give a better experimental result as well. In terms of visible effect, our results perform clearer edge and produce more high-frequency information than traditional SR method. Compared to the state-of-the-art SR method, our method by using adaptive multi-scale detail enhancement works better in handling those different scale edges. The reconstructed results produce clear edge structure and rich detail information. This strategy exploits the availability of rich remote-sensing image to assist the user in providing more high-resolution image.

In the real remote-sensing image experiments, the objective assessment is provided through the non-referenced image quality assessment method, due to the real HR images cannot be obtained. The non-referenced image quality assessment indices are entropy and EME, respectively. These two indices mainly evaluate the amount of information of the image. The higher the value, the better the image quality. The objective evaluation indexes are shown in Table 3, where the entropy and EME metrics by contrasting different methods on the remote-sensing images are listed. Statistics show that the proposed AMED-SR method achieves the highest Entropy and EME metrics on almost all the experimental images. The average entropy gains over bicubic, IBP, MAP, SRCNN, VDSR, HE and average fusion method are 0.84 dB, 0.34 dB, 0.31 dB, 0.32 dB, 0.3 dB, 0.57 dB and 0.27 dB, respectively. In contrast with the other SR methods, the EME metric of the proposed SR method is improved significantly. Table 3 shows the objective evaluation index of proposed method in this paper is better than the traditional SR methods and the state-of-the art SR methods. In addition, histogram equalization through gray stretching may lead to the entropy index slightly higher occasionally, but the detail information is not improved in experiment seven. Combined with the final image of the experiment, the visual effect is poor and it has missed part of the information. The entropy and EME metric of the weighted fusion is a little bit higher than average fusion. It reveals that AMED-SR method can provide rich detail information in the process of the super-resolution reconstruction by synthesizing subjective evaluation and objective evaluation analyses.

In this paper, we established a novel nonlinear detail-enhancement function. Using this function, texture-detail information of remote-sensing images is promoted. The SRCNN and VDSR method is the state-of-the-art SR method, and the performance of the SRCNN and VDSR model is based on deep learning networks. However, such deep learning methods exhibit limitations in terms of architecture, e.g., deep learning cannot realize adaptive scale SR reconstruction; therefore, methods based on deep learning can only train the specified model parameters. However, after experimental analysis, we found a better model with which to increase differently scaled information in remote-sensing images. It is clear that this model can realize SR reconstruction with different factors through the nonlinear detail-enhancement function, thereby avoiding training samples for a specific scale alone. Compared with traditional SR methods, such as the bicubic, IBP and MAP methods, the adaptive multi-scale detail-enhancement model delivers the best performance in the matter of improving the entropy. Our method improves on the traditional method because it cannot increase the high-frequency information, which leads to limited resolution of the reconstructed image. Traditional SR methods focus purely on the content of the image itself, while not focusing on texture details, which is the main difference between the traditional SR methods and the proposed model. In the future, we plan to combine radiation information to improve the effect of the reconstructed image. We are also interested in applying the radiation prior to the point-spread-function estimation as the constraint condition to make the reconstructed image clearer.

## 4. Conclusions

In this work, we have developed a novel remote-sensing image SR reconstruction method. In our method, through spatio-temporal remote-sensing data preprocessing, the differences between spatio-temporal remote-sensing data is reduced and the complementarity of spatio-temporal remote-sensing data is enhanced. Multi-scale non-redundant information is extracted, which is made full use of in the process of the SR reconstruction. At the same time, for the problem of remote-sensing image SR, the traditional SR methods will become less effective because they fail to improve the high-frequency detail information. We addressed this issue by using the adaptive nonlinear detail-enhancement function. Through multi-scale detail enhancement, AMDE-SR can act as a new method of increasing structural data and fidelity. Actual results show an average gain in luminance entropy with up to 0.42 dB for an up-scaling of 2. Real results show an average gain in EME with up to 4.25 dB for an up-scaling of 2. Experiments show that AMDE-SR can greatly increase high-frequency detail information, making remote-sensing image SR more effective. Furthermore, the proposed AMDE-SR method, compared with the state-of-the-art SR method, can reconstruct different zooming factors, instead of training different factor models, and the reconstructed HR images achieve state-of-the-art performance. Our extensive experimental results demonstrate that the proposed AMDE-SR method significantly outperforms state-of-the-art remote-sensing image SR methods in terms of both quantitative metrics and subjective visual quality.

## Figures and Tables

**Table 1:** The Objective Evaluation Index Results of Different SR Methods.

Image Data	Bicubic	IBP	SRCNN	Proposed
Experiment one	PSNR: 25.59	PSNR: 26.05	PSNR: 26.38	PSNR: 26.77
	SSIM: 0.82	SSIM: 0.85	SSIM: 0.87	SSIM: 0.89
Experiment two	PSNR: 20.57	PSNR: 22.03	PSNR: 26.43	PSNR: 26.83
	SSIM: 0.74	SSIM: 0.82	SSIM: 0.89	SSIM: 0.91
Experiment three	PSNR: 21.41	PSNR: 21. 82	PSNR: 22.41	PSNR: 22.52
	SSIM: 0.80	SSIM: 0.82	SSIM: 0.88	SSIM: 0.91
Experiment four	PSNR: 32.11	PSNR: 32.16	PSNR: 33.08	PSNR: 33.10
	SSIM: 0.81	SSIM: 0.86	SSIM: 0.94	SSIM: 0.95
Experiment five	PSNR: 29.96	PSNR:30.02	PSNR: 30.17	PSNR: 30.18
	SSIM: 0.85	SSIM: 0.89	SSIM: 0.92	SSIM: 0.94
Experiment six	PSNR: 29.94	PSNR: 30.06	PSNR: 30.15	PSNR: 30.17
	SSIM: 0.83	SSIM:0.87	SSIM: 0.90	SSIM: 0.91

**Table 2:** The parameters of experimental imagery.

No.	Figure	Satellite	View/Spectral Mode	Image Size	GSD (m)	Acquisition Date
1	7a	ZY3-01	Nadir-View	2000 × 2000	2.1	10 July 2013
		ZY3-01	Forward-View	2000 × 2000	3.5	10 July 2013
		ZY3-01	Backward-View	2000 × 2000	3.5	10 July 2013
2	7b	ZY3-01	Nadir-View	705 × 705	2.1	9 February 2016
		ZY3-01	Nadir-View	705 × 705	2.1	3 April 2016
		ZY3-01	Nadir-View	705 × 705	2.1	8 April 2015
3	7c	ZY3-01	Nadir-View	500 × 500	2.1	30 January 2016
		ZY3-01	Nadir-View	500 × 500	2.1	4 February 2016
		ZY3-01	Nadir-View	500 × 500	2.1	29 March 2016
4	7d	ZY3-01	Nadir-View	500 × 500	2.1	30 January 2016
		ZY3-01	Nadir-View	500 × 500	2.1	24 March 2016
		ZY3-01	Nadir-View	500 × 500	2.1	29 March 2016
5	7e	GF-2	Panchromatic	500 × 500	0.8	3 November 2017
		GF-2	Panchromatic	500 × 500	0.8	11 November 2017
		GF-2	Panchromatic	500 × 500	0.8	7 December 2017
6	7f	GF-2	Multi Spectral	500 × 500	3.2	11 November 2017
7	7h	ZY3-01	Nadir-View	500 × 500	2.1	17 May 2016
		ZY3-02	Nadir-View	500 × 500	2.1	5 June 2016
		ZY3-02	Forward-View	500 × 500	3.5	5 June 2016

**Table 3:** Entropy and EME Values of Different Reconstruction Methods in Real Experiments.

	Bicubic	IBP	MAP	SRCNN	VDSR	HE	Average Fusion	Proposed
Exp_1	Entropy:6.18	Entropy:6.26	Entropy:6.21	Entropy:6.28	Entropy:6.29	Entropy:6.11	Entropy:6.46	Entropy:7.01
	EME:5.93	EME:6.05	EME: 5.34	EME:6.17	EME:6.54	EME:6.80	EME:12.26	EME:14.47
Exp_2	Entropy:6.89	Entropy:7.09	Entropy: 7.10	Entropy:7.10	Entropy:7.12	Entropy:7.06	Entropy:7.10	Entropy:7.56
	EME:8.41	EME:9.05	EME: 9.18	EME:9.13	EME:9.66	EME:10.67	EME:14.87	EME:15.15
Exp_3	Entropy:6.95	Entropy:6.96	Entropy: 6.98	Entropy:6.93	Entropy:6.97	Entropy:6.83	Entropy:6.92	Entropy:7.12
	EME:10.08	EME:10.11	EME: 11.81	EME:11.88	EME:11.87	EME:12.28	EME:12.63	EME:13.07
Exp_4	Entropy:6.62	Entropy:6.63	Entropy: 6.75	Entropy:6.78	Entropy:6.97	Entropy:6.78	Entropy:6.90	Entropy:7.18
	EME:4.69	EME:4.79	EME: 6.42	EME:7.23	EME:8.71	EME:6.94	EME:8.55	EME:9.44
Exp_5	Entropy:6.09	Entropy: 7.15	Entropy: 7.14	Entropy:7.16	Entropy:7.11	Entropy:7.28	Entropy: 7.24	Entropy:7.46
	EME:5.82	EME:7.19	EME: 5.70	EME:7.80	EME:6.23	EME:9.34	EME: 11.03	EME: 12.75
Exp_6	Entropy:6.54	Entropy:7.57	Entropy: 7.60	Entropy:7.56	Entropy: 7.60	Entropy:5.95	Entropy:7.56	Entropy:7.58
	EME:8.03	EME:8.85	EME: 8.86	EME:8.87	EME:8.61	EME:7.78	EME:13.63	EME:13.99
Exp_7	Entropy:6.45	Entropy:7.54	Entropy:7.62	Entropy:7.58	Entropy: 7.45	Entropy:7.72	Entropy:7.51	Entropy:7.56
	EME:4.63	EME:4.64	EME:4.99	EME:5.55	EME:8.03	EME:6.34	EME:8.64	EME:9.30
